# Creation and Validation of a Survival Nomogram Based on Immune-Nutritional Indexes for Colorectal Cancer Patients

**DOI:** 10.1155/2022/1854812

**Published:** 2022-03-25

**Authors:** Yulan Liu, Yang Meng, Chenliang Zhou, Ya Liu, Shan Tian, Jiao Li, Weiguo Dong

**Affiliations:** ^1^Department of Critical Care Medicine, Renmin Hospital of Wuhan University, Wuhan, China; ^2^Department of Gastrointestinal Surgery II, Renmin Hospital of Wuhan University, Wuhan, China; ^3^Department of Gastroenterology, Renmin Hospital of Wuhan University, Wuhan, China; ^4^Department of Infectious Diseases, Union Hospital, Tongji Medical College, Huazhong University of Science and Technology, Wuhan, China

## Abstract

Nutritional and inflammatory status was associated with prognosis in various types of malignant cancer, including colorectal cancer (CRC). This clinical research was performed to estimate the prognostic role of immune-nutritional indexes CRC in patients and to set up a survival nomogram based on the significant immune-nutritional indexes. 1024 CRC patients underwent surgical resection from Wuhan Union Hospital were enrolled and divided into the test cohort (*n* = 717) and validation cohort (*n* = 307). A total of 19 immune-nutritional indexes were included into our analysis. The Cox regression analysis was utilized to identify the informative immune-nutritional indexes which were closely associated with overall survival (OS) and disease-free survival (DFS). Survival nomograms were created in the test set and further verified in the validation set. Td-ROC was curved to estimate the predictive performance of survival nomograms for CRC patients. Body mass index (BMI), chemotherapy, TNM stage, T stage, lactate dehydrogenase (LDH)/prealbumin (PA), monocytes (MON)/albumin (ALB), and prognostic nutritional index (PNI) were seven potent prognostic biomarkers of CRC patients. We created an OS-nomogram based on the seven risk indexes, and the predictive accuracy expressed with area under curve (AUC) was 0.826 for 1-year, 0.809 for 3-year, and 0.80 for 5-year OS rates in the test set and 0.795 for 1-year, 0.749 for 3-year, and 0.647 for 5-year OS rates in the validation set. TNM stage, T stage, LDH/ALB, and MON/ALB were risk factors for unfavorable DFS in CRC patients. We further built a DFS-nomogram based on the four risk factors, and the predictive performance presented with AUC was 0.806 for 1-year, 0.763 for 3-year, and 0.82 for 5-year DFS rates in the test set, and 0.704 for 1-year, 0.692 for 3-year, and 0.692 for 5-year DFS rates in the validation set. Our survival nomogram based on immune-nutritional indexes is a useful and potential prognostic tool in CRC patients.

## 1. Introduction

Colorectal cancer (CRC) accounted for 12.7% of all newly diagnosed cancer, which is the second most frequently occurring cancer [1]. In 2020, CRC accounted for 12.4% of all deaths, being the second most common cause of cancer death based on data from 27 countries of the European Union [2]. The exact pathogenic mechanism of CRC is still uncertain, but genetic susceptibility, gut flora, dietary habit, and environmental factors are reported to play key roles in its occurrence [3]. The mainstream treatment for CRC is based on comprehensive approaches, composed of surgery, radiation, chemotherapy, and emerging immunotherapy. Although curative removal of the tumor tissues is expected to be a curative treatment for CRC, the long-term survival outcome of CRC patients is still not promising due to the early recurrence.

Inflammation and malnutrition are proven to be involved in the progression of CRC [4]. Systemic inflammation is a marker of worse survival outcomes in approximately 20%-40% of CRC patients [5]. Several clinical studies have highlighted that serum inflammatory indexes, such as systemic immune-inflammation index (SII), pan-immune-inflammation (PII) [6], controlling nutritional status score (CONUT) [7], Gustave Roussy Immune (GRIm) Score [8], could well forecast the survival outcomes of CRC individuals. Moreover, CRC patients are the high-risk population with malnutrition, which is associated with impaired therapeutic response and higher mortality [9]. Continued malnutrition is more common in CRC patients with advanced cancer and could speed up to early death in the condition of no effective nutrition support. Hence, a better understanding of CRC patient's immune-nutritional status is critical to their survival outcomes.

Immune-nutritional indexes could not only reflect the inflammatory status of the body but also reflect the nutritional condition. Hence, early identification of inflammation and malnutrition in CRC patients is crucial. However, clinicians tend to belittle this phenomenon in the clinical practice, making it very imperative to assess the inflammatory and nutritional status of CRC patients. Among these indexes, PNI, CONUT, and GRIm scores are reported to well reflect the host immune-nutrition status in CRC patients. As immune-nutritional indexes are inexpensive to test for blood and easily accessible in the clinical practice, it is quite significant to identify novel immune-nutritional indexes for the assessment of survival outcomes in CRC patients. Hence, in this present study, our primary goal was to assess the prognostic significance of a list of novel immune-nutritional indexes. Then, our second goal was to derive and verify two survival nomograms based on immune-nutritional indexes for the precise prediction of survival outcomes in CRC patients.

## 2. Materials and Methods

### 2.1. Study Population

A total of 1474 CRC sufferers from Wuhan Union Hospital were initially analyzed, and only 1024 cases of CRC were included into the final analysis. The inclusion criteria were listed as follows: (1) the diagnosis of CRC confirmed by pathological reports, (2) surgical management performed as the first treatment, (3) CRC patients with complete preoperative laboratory examination information, and (4) CRC patients with no evidence of acute infection. The exclusion criteria were as follows: (1) systemic chemotherapy or radiotherapy before surgical resection, (2) CRC patients under the age of 18 years, (3) CRC patients who lost for follow-up, (4) CRC patients were complicated with obvious acute infection, and (5) administration of anti-inflammatory agents prior to the initiation of the surgery, such as antibiotics, nonsteroidal anti-inflammatory drugs, and glucocorticoid. All relevant materials were checked and approved by the Clinical Research Ethics Committee (CREC) of Wuhan Union Hospital (No. 2018-S377). Written informed consents were obtained from all participants prior to the initiation of this clinical research.

### 2.2. Data Collection

We retrospectively collected CRC patients' baseline data and clinical information before surgical management, including demographic data, clinical information, and laboratory data. The demographic data were composed of sex, age of diagnosis, and body mass index (BMI). The clinical information was composed of tumor size, tumor site, T stage, N stage, tumor differentiation, and TNM stage. The laboratory data were composed of blood routine [lymphocyte (LYM), neutrophil (NEU), monocyte (MON), and platelet (PLT)], liver function [albumin (ALB), prealbumin (PA), lactate dehydrogenase (LDH), alkaline phosphatase (ALP), and glutamyltransferase (GGT)], and renal function [creatinine (CREA)]. Moreover, we calculated the novel immune-nutritional indexes, such as LDH/PA, LDH/ALB, GGT/ALB, GGT/PA, ALP/ALB, ALP/PA, PLT/ALB, PLT/PA, LYM/ALB, LYM/PA, NEU/ALB, NEU/PA, MON/ALB, MON/PA, ALB/CREA, and PA/CREA. The cutoff values of these immune-nutritional indexes were determined by X-tile software (version 3.4.7). We also included three established immune-nutritional indexes, PNI, CONUT, and GRIm score. The GRIm score was obtained according to a previous study [8] based on serum lactate dehydrogenase, serum albumin, and NLR.

### 2.3. Development and Validation of Survival Nomogram

In order to derive and verify a survival nomogram with robustness, we randomly assigned the included CRC patients into the test set (*N* = 717) and validation set (*N* = 307) according to the ratio of 7 : 3. In the test set, we first developed an overall survival nomogram (OS-nomogram). We initially employed a univariate Cox regression to identify the immune-nutritional variables with a close relationship to OS in CRC patients. Then, the significant immune-nutritional metrics with *P* < 0.05 were further selected into multivariate Cox regression. Only the immune-nutritional indexes determined by multivariate Cox regression (*P* < 0.05) were finally identified for the construction of OS-nomogram. The risk score equation behind the OS-nomogram was determined using the *β*-coefficients of the multivariate Cox regression analysis. To validate the OS-nomogram, the predicted OS rates of CRC patients in the internal validation cohort were also measured using the same regression equation derived from the test set. Similarly, the DFS nomogram was constructed based on the same method. The discrimination ability of survival nomogram for predicting survival rate was measured by time-dependent (td) receiver operating characteristic (ROC) curves. Each survival nomogram was assessed with calibration curve, which made it possible to compare the predicting survival rates with the actual survival rates. We also verified the discrimination and calibration abilities of the two survival nomograms in the validation cohort. Finally, decision curve analysis (DCA) was drawn to appraise the clinical utility of the survival nomograms. DCA is a statistical method which is widely used to evaluate prediction models. DCA attempted to overcome the limitations of discrimination and calibration which are not very informative to full decision analytic approaches. DCA compares a clinical “net benefit” for a predictive model with default strategies of none treating or treating [10].

### 2.4. Statistical Analysis

All the statistical analyses were implemented with SPSS (version 20.0), MedCalc application (version 19.0.4), and R software (version 3.5.1). Categorical indexes were presented with counts (*n*) and percentages (%) and examined by a chi-square test or Fisher's exact. Continuous variables were expressed as the mean differences and standard deviation or interquartile range (IQR) based on the status of data distribution. Continuous data were analyzed with Student's *t*-test or nonparametric test. Spearman's correlation analysis was adopted to measure the relationship between two immune-nutritional indexes [11]. The cumulative survival rates of CRC patients were estimated by survival analysis and analyzed using the log-rank test. Univariate combined with multivariate Cox analyses were performed to evaluate the overall effects of included variables on the survival outcomes of CRC patients. Td-ROC curves were plotted to determine the prediction accuracy of the inflammatory indexes or survival nomogram for 1-year, 3-year, and 5-year survival rates. The Akaike information criterion (AIC) was also calculated to assess the goodness of fit of the survival nomograms. AIC analysis was viewed as a good statistical system for the identification of predictive markers, which offer statistical significance for the balance between complexity and adaptation of a predictive model. AIC quantifies the relative goodness of fit for various metrics for a preferred model. The predictive model with the lowest AIC value is considered the preferred model, and the lower the AIC, the better the predictive model [12]. A *P* value less than 0.05 signifies that the difference is significant.

## 3. Results

### 3.1. Clinical Features of Included Participants

**Figure 1:**
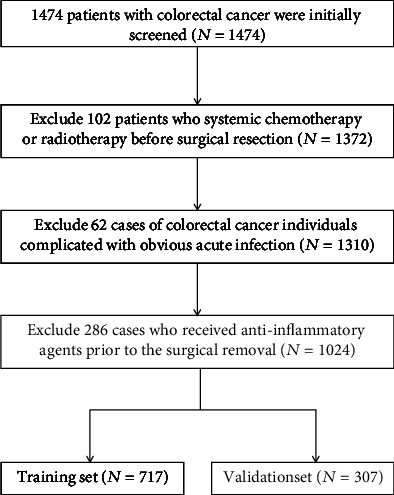
Flow chart of participant selection.

According to the strict inclusion criteria, a total of 1024 CRC patients who underwent surgical removal were screened into our analysis (Figure 1). Among them, the majority of participants were men (60.45%), and the median age of these included CRC patients was 58.399 ± 11.87 years. Among all, 4.21% and 63.27% of patients had a low body weight (LBW) and normal BMI, respectively. All the included patients received surgical resection, and 541 cases of CRC patients received postoperative chemotherapy.

### 3.2. Correlations among Immune-Inflammation Indexes

**Figure 2:**
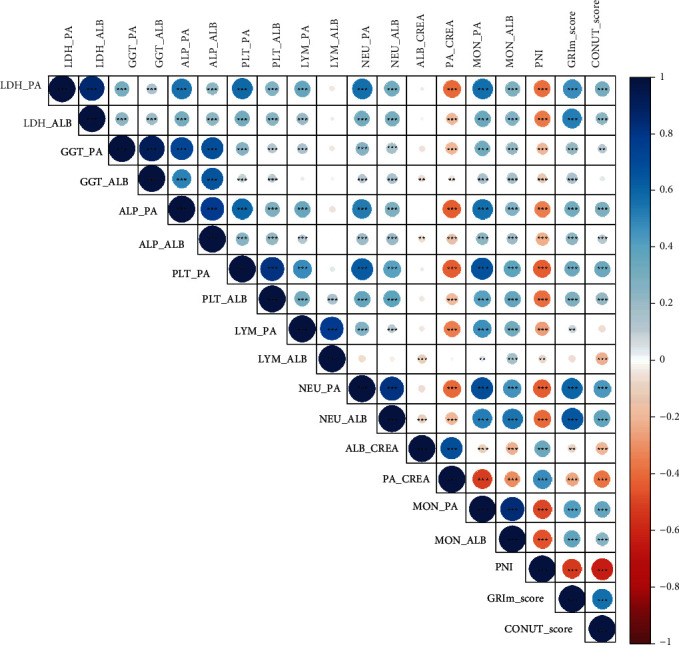
Correlations of each immune-inflammation index in CRC.

In this study, we systematically assessed all the available immune-inflammation variables in CRC patients. A total of 19 immune-inflammation indexes were included into our analysis. Based on the correlation analysis, we found these immune-inflammation indexes correlated with each other. As listed in Figure 2, we observed that LDH/PA was strongly correlated with LDH/ALB (*r* = 0.77, *P* < 0.0001), ALP/PA (*r* = 0.68, *P* < 0.0001), PLT/PA (*r* = 0.62, *P* < 0.0001), NEU/PA (*r* = 0.61, *P* < 0.0001), MON/PA (*r* = 0.57, *P* < 0.0001), and GRIm score (*r* = 0.49, *P* < 0.0001), but reversely associated with PA/CREA (*r* = −0.56, *P* < 0.0001). As for LDH/ALB, although this score was correlated with many other immune-inflammation biomarkers, the correlation was less significant than LDH/PA.

### 3.3. Overall Survival Nomogram Based on Immune-Nutritional Indexes

**Figure 3:**
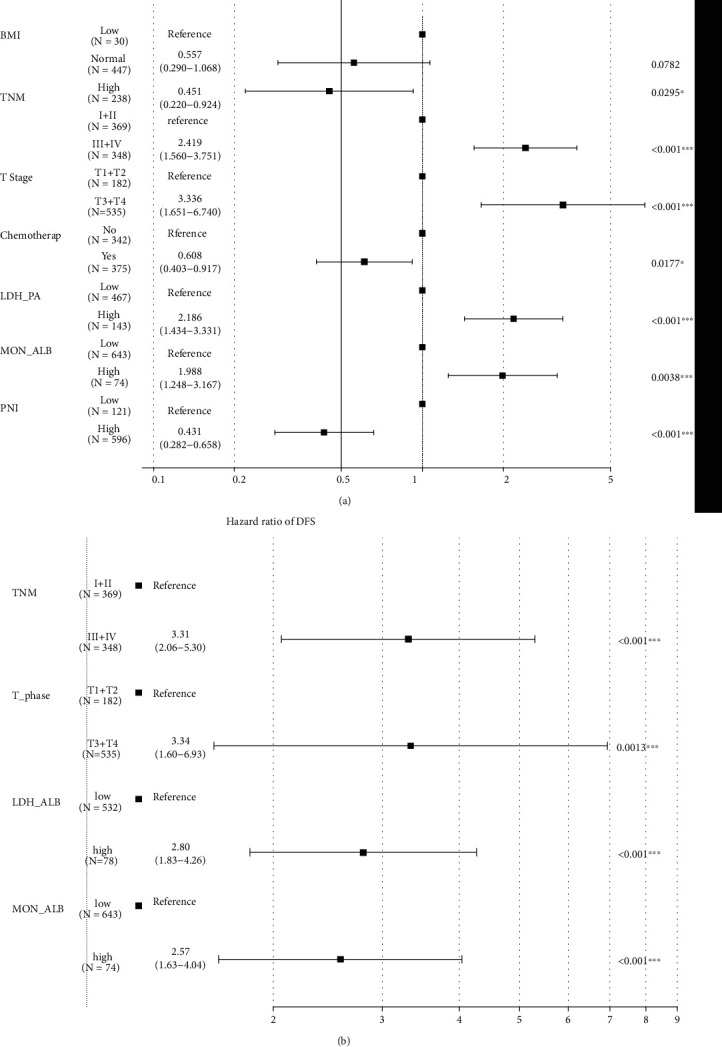
Multivariate Cox regression of survival outcomes in individuals with CRC. (a) Overall survival. (b) Disease-free survival.

For the purpose of building survival model based on immune-nutritional indexes, we randomly assigned these CRC individuals into test set (*N* = 717) and validation set (*N* = 307) according to the ratio of 7 : 3. There were no significant differences of clinical features in the test and validation sets (Table S1). In the test set, we initially utilized univariate Cox analysis to estimate the potential risk indexes which could significantly influence the OS in CRC patients. We identified that 24 significant features, including BMI, TNM stage, T stage, N stage, tumor size, chemotherapy, LDH/PA, LDH/ALB, GGT/PA, GGT/ALB, ALP/PA, ALP/ALB, PLT/PA, PLT/ALB, LYM/PA, NEU/PA, NEU/ALB, ALB/CREA, PA/CREA, MON/PA, MON/ALB, PNI, GRIm score, and CONUT score, were all well correlated with the OS in CRC patients (Table S2). Then, these informative immune-inflammation indexes with *P* < 0.05 were further selected into the multivariable Cox model. We noticed that BMI (HR = 0.451, 95% CI: 0.022-0.924, *P* = 0.0295), chemotherapy (HR = 0.608, 95% CI: 0.403-0.917, *P* = 0.0177), T stage (HR = 3.336, 95% CI: 1.651-6.74, *P* < 0.001), TNM stage (HR = 2.419, 95% CI: 1.560-3.751, *P* < 0.001), LDH/PA (HR = 2.186, 95% CI: 1.434-3.331, *P* < 0.001), MON/ALB (HR = 1.988, 95% CI: 1.248-3.167, *P* < 0.001), and PNI (HR = 0.431, 95% CI: 0.282-0.658, *P* < 0.001) were still potent prognostic biomarkers of CRC patients after adjusting the confounding covariates (Figure 3(a)).

**Figure 4:**
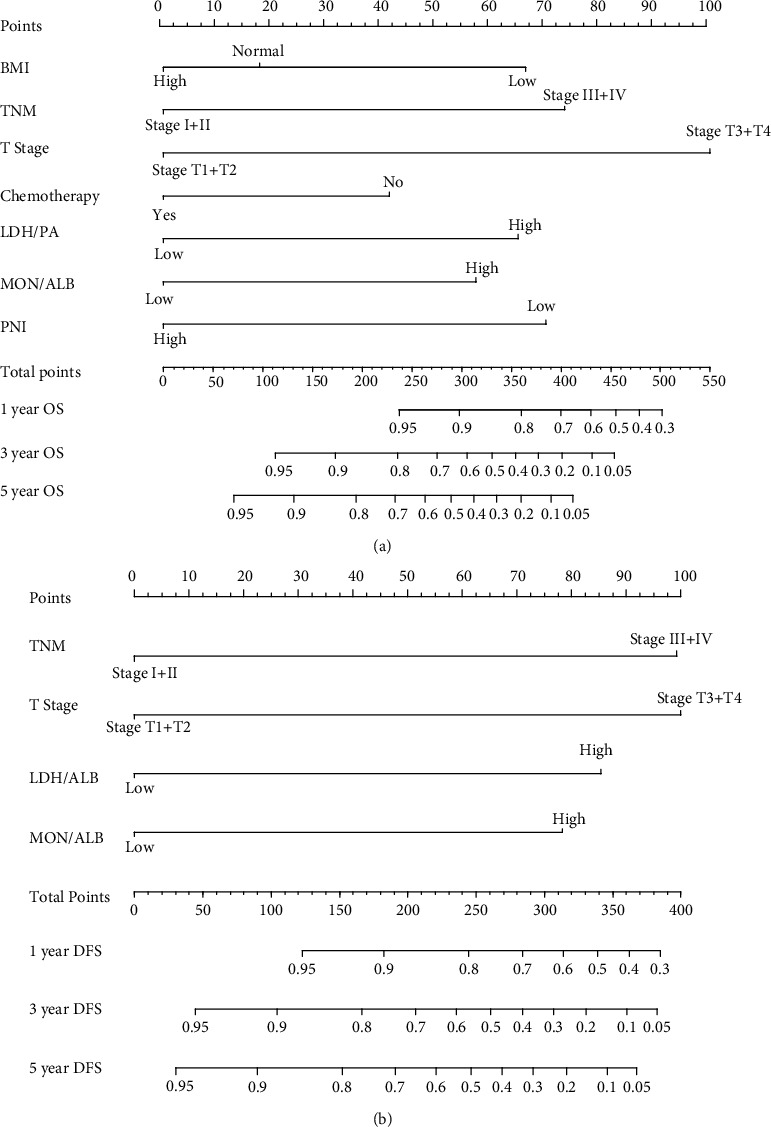
Survival nomograms based on immune-inflammation indexes for the prediction of CRC patients' survival mortality. (a) Overall survival nomogram. (b) Disease-free survival nomogram.

**Figure 5:**
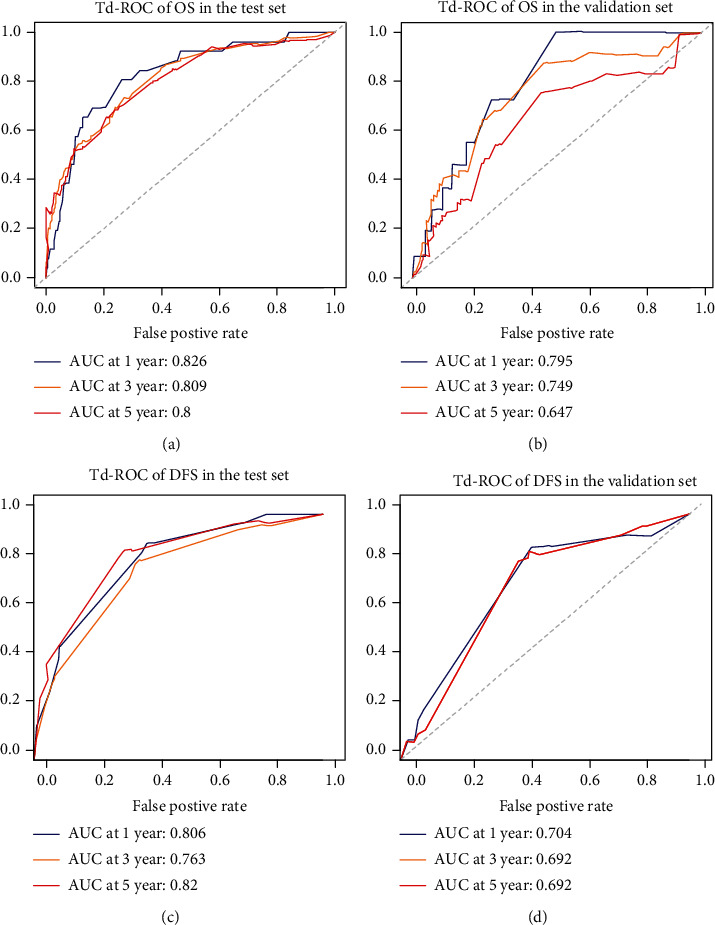
Predictive accuracy of survival nomograms presented with td-ROC curves. (a) Prediction of overall survival rate in the test set. (b) Prediction of overall survival rate in the validation set. (c) Prediction of disease-free survival rate in the test set. (d) Prediction of disease-free survival rate in the validation set.

**Figure 6:**
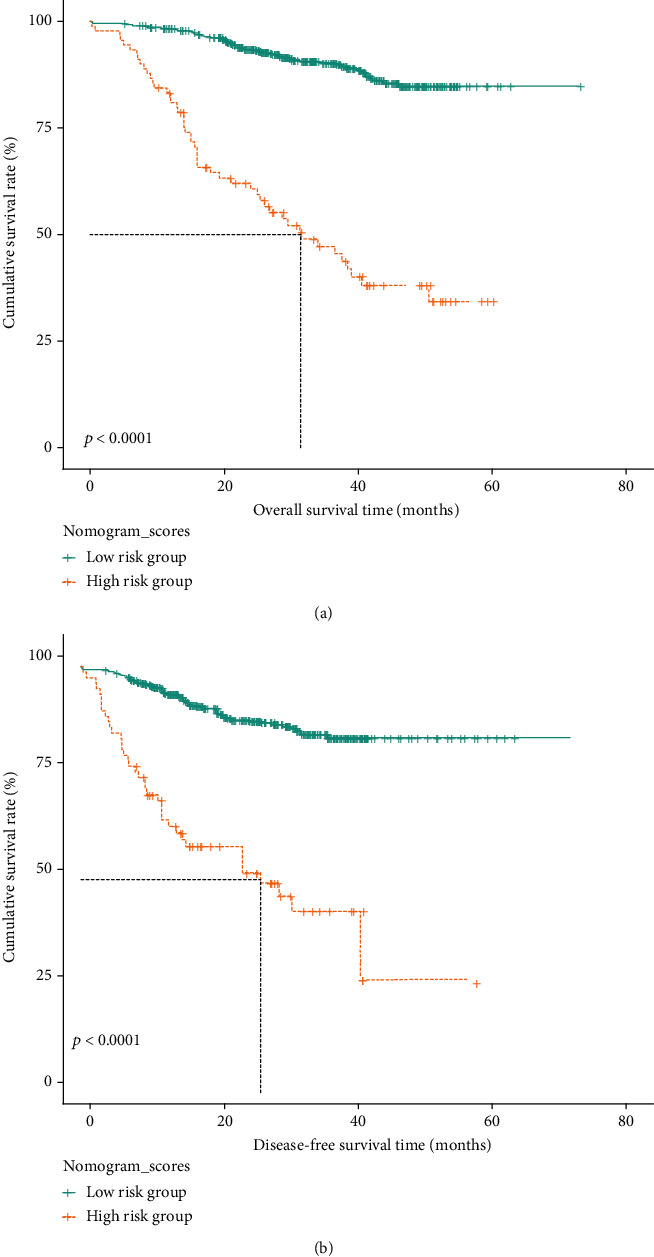
Kaplan-Meier curves of survival nomograms by two groups. (a) Overall survival analysis. (b) Disease-free survival analysis.

Hence, we further created an OS-nomogram based on the seven risk indexes (Figure 4(a)), and the predictive accuracy expressed with AUC was 0.826 for 1-year, 0.809 for 3-year, and 0.80 for 5-year survival rates in the test set (Figure 5(a)). This OS-nomogram exhibited the smallest AIC value of 1131. When validating the OS-nomogram in validation set, the predictive accuracy expressed with AUC was 0.795 for 1-year, 0.749 for 3-year, and 0.647 for 5-year survival rates in CRC patients (Figure 5(b)). Moreover, we divided these CRC patients into two categories (low risk and high risk) based on the median value of OS-nomogram, and the Kaplan-Meier curves exhibited the great survival difference between the two groups (*P* < 0.001), highlighting the great value of the OS-nomogram for risk stratification of CRC patients (Figure 6(a)).

### 3.4. Disease-Free Survival Nomogram Based on Immune-Nutritional Indexes

Similarly, univariate analysis revealed that TNM stage, T stage, N stage, chemotherapy, LDH/PA, LDH/ALB, GGT/PA, GGT/ALB, ALP/PA, ALP/ALB, PLT/PA, PLT/ALB, LYM/ALB, NEU/PA, NEU/ALB, ALB/CREA, PA/CREA, MON/PA, MON/ALB, PNI, GRIm score, and CONUT score were all well correlated with the DFS in CRC patients (Table S2). After adjusting the confounding covariates, TNM stage (HR = 3.31, HR = 2.06 − 5.30, *P* < 0.001), T stage (HR = 3.34, HR = 1.60‐6.93, *P* = 0.0013), LDH/ALB (HR = 2.80, 95% CI: 1.83-4.26, *P* < 0.001), and MON/ALB (HR = 2.57, 95% CI: 1.63-4.04, *P* < 0.001) were risk factors for unfavorable DFS in CRC patients (Figure 3(b)). Hence, we further built a DFS-nomogram based on the four risk factors (Figure 4(b)), and the predictive performance expressed with AUC was 0.806 for 1-year, 0.763 for 3-year, and 0.82 for 5-year survival rates in the test set (Figure 5(c)). This DFS-nomogram exhibited the smallest AIC value of 1137.8. When verifying the DFS-nomogram in the validation set, the predictive performance presented with AUC was 0.794 for 1-year, 0.692 for 3-year, and 0.692 for 5-year survival rates in CRC patients (Figure 5(d)). Additionally, we also divided these CRC patients into two categories (low risk and high risk) based on the median value of DFS-nomogram, and the survival analysis exhibited the distinct survival difference among the two groups (*P* < 0.001), highlighting the potential value of the DFS-nomogram for risk stratification of CRC patients (Figure 6(b)).

### 3.5. Calibration Ability and Clinical Utility of Survival Nomograms

**Figure 7:**
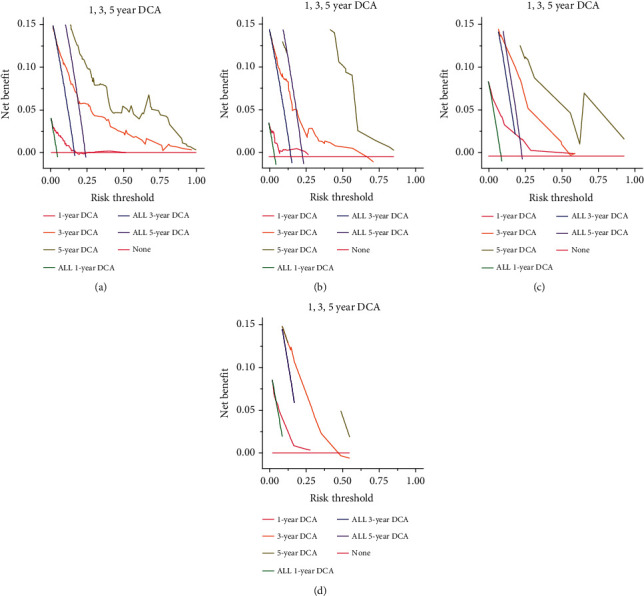
The clinical utility of survival nomograms by decision curve analysis (DCA). (a) DCA of overall survival in the test set. (b) DCA of overall survival in the validation set. (c) DCA of disease-free survival in the test set. (d) DCA of disease-free survival in the validation set.

We applied the calibration curves to compare actual probabilities of survival rates and the predicted survival rates by survival nomograms. Figure S1A-F demonstrates good agreement for 1-year, 3-year, and 5-year predicting probabilities of OS rates and actual survival rates in both test and validation cohorts. In addition, Figure S2A-F also shows good agreement for the 1-year, 3-year, and 5-year predicting probabilities of DFS rates and actual survival rates in both test and validation sets. Moreover, we employed DCA to assess the clinical utilities of the survival nomograms for CRC patients. As presented in Figures 7(a) and 7(b), if the threshold probability of a patient was 0.25, both the OS-nomogram and DFS-nomogram added more clinical benefits than either treat-none scheme or treat-all scheme, implicating that the survival nomograms were clinically applicable for CRC patients.

## 4. Discussion

Systemic inflammation and malnutrition is prevailing in patients with cancer. The two factors have a significant impact on the quality of life and treatment outcomes in cancer population [13]. As malnutrition is a major element for immunodeficiency, the nutritional condition can be used to quickly evaluate the immune status of cancer patients [14]. Some clinical cohorts and meta-analyses investigated the associations between the immune-inflammation index evaluated by laboratory data and survival outcomes in malignant cancers [15–18], but few clinical researches have appraised this correlation in CRC patients. This clinical research systematically assessed the available immune-inflammation indexes (*N* = 19) as many as possible in CRC patients. Then, we found that LDH/PA, LDH/ALB, PNI, and MON/ALB possess the most outstanding performance in the prediction of survival outcomes, and we also measured its correlations with other immune-inflammation indexes. Finally, we screened the most informative immune-inflammation elements based on Cox regression for the construction survival nomograms. Both OS and DFS nomograms derived from immune-inflammation parameters exhibited adequate discrimination and well clinical utility.

Serum LDH usually converts pyruvate to lactate in the condition of hypoxia, which occupies an important role in the metabolism of tumor cells. LDH-A is reported to be highly expressed in metastatic cancer cells and hypoxic carcinomas, whose levels closely associated with the viability of cancer cells. Levels of serum LDH are markers of immune suppression and tumor hypoxia [19–21]. Moreover, recent studies also revealed that high levels of serum LDH signify heavy tumor burden and tumor progression in cancer [22, 23]. Hence, we could conclude that high levels of serum LDH are indicative of unfavorable survival outcomes in cancer individuals. As mentioned above, serum albumin level could well reflect the nutritional status of cancer patients, and tumor-related inflammatory response may contribute to the loss of albumin. In LDH/ALB, a novel immune-inflammation biomarker, its high level means severe inflammation and worse nutritional status. LDH/ALB is reported to be highly correlated with survival outcomes in some types of tumors, but fewer studies explored its correlation with the survival outcomes in CRC patients.

Feng et al. [24] conducted a retrospective study with a cohort of 346 resectable esophageal squamous cell carcinoma (ESCC) and concluded that LDH/ALB is a useful prognostic biomarker in patients with resectable ESCC who received surgical resection. Gan et al. [25] assessed the prognostic role of serum LDH/ALB in a cohort of 1,041 liver cancer patients who received curative resection, and they demonstrated that serum LDH/ALB was superior to other inflammatory scores in terms of predicting survival in liver cancer individuals who underwent radical surgical removal. A cohort study from Turkey including 295 cases of CRC patients also reached the similar conclusion that preoperative LDH/ALB was an unfavorable prognosticator in CRC patients receiving curative resection [26]. However, the sample size (*N* = 295) somewhat limited the persuasion of the conclusion. In our study (*N* = 1024), we also found the superior predictive performance of serum LDH/ALB, and serum LDH/ALB was not only a strong prognostic biomarker for unfavorable OS but also an independent risk element for inferior DFS in CRC patients.

Protein-related malnutrition is very common in cancer patients with advanced stage and eventually leads to the damage of immune barrier. Malnutrition can seriously affect the biosynthesis of PA and ALB [27]. Compared with ALB, PA has a shorter half-life (2 days) than ALB (12 days) and could be utilized as a promising marker to monitor the nutritional status. Our study not only explored the prognostic role of serum LDH/ALB in CRC patients but also assessed the prognostic significance of serum LDH/PA in CRC patients. The multivariate Cox regression analysis revealed that serum LDH/PAB was a potent risk factor for inferior OS and DFS in patients with CRC.

The application of Mon/ALB is an objective assessment criterion of inflammatory and nutritional status, which is completely based on easily available laboratory parameters. Monocyte count, directly from blood routine, is a direct parameter of inflammatory response and also reflects the condition of immune surveillance to tumor cells. In a multicenter study with 1052 cases of rectal cancer patients, Fulop et al. [28] highlighted the clinical significance of lymphocyte-to-monocyte ratio. They clarified that the lymphocyte-to-monocyte ratio was inversely associated with neutrophil-to-lymphocyte, and the preoperative values of lymphocyte-to-monocyte can be utilized as an independent risk biomarker for less unfavorable OS in rectal cancer individuals. Serum ALB can not only effectively reflect the nutritional status of cancer patients but also be related to the severe liver function caused by inflammatory cytokines [29]. Our results confirmed that CRC patients with high Mon/ALB were more likely to experience worse OS and DFS. Compared with some established immunonutritional indexes commonly applied in clinical practice, such as GRIm score and CONUT, MON/ALB is more accurate and convenient for immune-inflammation evaluation in patients with CRC.

Our aim was to design a precise survival model based on the independent prognostic factors for patients with CRC. Since the endpoints of this analysis were OS status, OS time, DFS status, and DFS time, so we selected the Cox proportional hazards model rather than the Kaplan-Meier marginal regression model. As the survival outcomes of CRC individuals are usually related to multiple endpoints which compete with one another to produce competitive risk data [30], the Cox proportional hazards model is a classical statistical model and widely employed in survival analysis for individuals with cancer. Accurate estimation of the cumulative incidence of survival outcomes for right-censored survival variables with multiple endpoints is the main advantage of Cox proportional hazards model. Hence, we identified seven potent factors for unfavorable OS and four risk factors for less favorable DFS in the test set based on the Cox proportional hazard model.

Three limitations still existed in this clinical analysis. First, this was a retrospective clinical research with relatively small study population. Then, we could not assess the association between dynamic changes of immune-nutritional indexes and survival outcomes in patients with CRC. In spite of the internal validation with 307 CRC patients, no external validation from another medical center was performed to evaluate the universal applicability of the survival nomograms. Hence, our conclusions should be further validated with prospective studies of more medical centers in the future.

## 5. Conclusion

This is the first scoring system based on immune-inflammation indexes to forecast survival outcomes in CRC sufferers. Notably, these selected immune-inflammation indexes are commonly tested among hospitalized patients in the clinical practice, which possess a practical advantage. This reliable predictive tool may play a role in risk stratification of CRC patients.

## Data Availability

The original contributions presented in the study are included in the article/Supplementary Material.

## Abbreviation

CRC: Colorectal cancer
OS: Overall survival
DFS: Disease-free survival
BMI: Body mass index
LDH: Lactate dehydrogenase (LDH)
PA: Prealbumin
ALP: Alkaline phosphatase
GGT: Glutamyltransferase
CREA: Creatinine
ALB: Albumin
MON: Monocytes
PLT: Platelet
LYM: Lymphocyte
NEU: Neutrophil
PNI: Prognostic nutritional index
GRIm: Gustave Roussy Immune
CONUT: Controlling nutritional status score
DCA: Decision curve analysis
AIC: Akaike information criterion
Td-ROC: Time-dependent receiver operating characteristic.

## Supplementary Materials

Supplementary Materials Table S1: the differences of basic features between test set and validation set. Table S2: univariate Cox regression of survival outcomes of CRC patients in the test cohort. Figure S1: calibration curves of OS-nomogram in the test (A-C) and validation cohorts (D-F). Figure S2: calibration curves of DFS-nomogram in the test (A-C) and validation cohorts (D-F).
