# Supplementary material for: Creation and Validation of a Survival Nomogram Based on Immune-Nutritional Indexes for Colorectal Cancer Patients
Source: J Oncol. 2022 Mar 25;2022:1854812. doi: 10.1155/2022/1854812 (PMC8975631; doi:10.1155/2022/1854812)
Supplement: Supplementary Materials — Table S1: the differences of basic features between test set and validation set. Table S2: univariate Cox regression of survival outcomes of CRC patients in the test cohort. Figure S1: calibration curves of OS-nomogram in the test (A-C) and validation cohorts (D-F). Figure S2: calibration curves of DFS-nomogram in the test (A-C) and validation cohorts (D-F). [file 1854812.f1.pdf]

Table S1. The differences of basic features between test set and validation set

Clinical features	Test set （n=717）	Validation set (n=307)	X^2/t value	P value
Sex			0.1294	0.7190
Male	436(60.81%)	183(59.61%)		
Female	281(39.19%)	124(40.39%)		
Age			0.3681	0.5441
<65	486(67.78%)	214(69.71%)		
>=65	231(32.22%)	93(30.29%)		
BMI			0.6502	0.7225
⩽18	30(4.2%)	13(4.25%)		
18-24	447(62.52%)	199(65.03%)		
⩾24	238(33.29%)	94(30.72%)		
Differentiation			0.2647	0.8760
Low	114(15.90%)	45(14.66%		
Moderate	551(76.85%)	240(78.18%)		
High	52(7.25%)	22(7.17%)		
TNM stage			4.5908	0.2043
I	110(15.34%)	38(12.38%)		
II	259(36.12%)	98(31.92%)		
III	275(38.35%)	136(44.30%)		
IV	73(10.18%)	35(11.40%)		
T_stage			1.8404	0.6062
T1	52(7.25%)	23(7.49%)		
T2	130(18.13%)	45(14.66%)		
T3	389(54.25%)	173(56.35%)		
T4	146(20.36%)	66(21.50%)		
N_stage			2.5057	0.2857
N0	419(58.44%)	163(53.09%)		
N1	183(25.52%)	88(28.66%)		
N2	115(16.04)%	56(18.24%)		
Location			3.0696	0.2155
Right	197(27.48%)	101(32.90%)		
Left	145(20.22%)	58(18.89%)		
Rectum	375(52.30%)	148(48.21%)		
Size			0.1693	0.6807
<5 cm	472(65.83%)	198(64.50%)		
≥5cm	245(34.17%)	109(35.50)%		
Chemotherapy			0.2704	0.6031
No	342(47.70%)	141(45.93%)		
Yes	375(52.30%)	166(54.07%)		
PLT	235.3±85.305	235.4±89.1981	-0.03	0.9782
MON	0.4535 ± 0.1768	0.4519±0.2019	0.12	0.9051

LYM	1.5569±0.9144	1.5305±0.5631	0.56	0.5738
NEU	4.1885±2.1157	4.2285±2.4830	-0.25	0.8054
ALB	40.09±4.7924	39.9629±4.9417	0.4	0.6911
ALP	78.5258±26.4184	78.4072±27.3122	0.07	0.9480
PA	0.2032±0.0609	0.1985±0.0621	1.13	0.2584
LDH	189.5±71.236	188.7±43.0716	0.19	0.8479
GGT	28.3668±32.8259	26.5277±30.8674	0.84	0.4033
CREA	71.4654±20.7272	71.1236±17.7893	0.27	0.7895

Table S2. Univariate Cox regression of survival outcomes of CRC patients in the test cohort.

Clinical factor	Overall survival		Disease-free survival	
	HR&95%CI	P value	HR&95%CI	P value
Gender	0.96 (0.67 - 1.4)	0.82	0.86 (0.6 - 1.2)	0.43
Age	0.98 (0.67 - 1.4)	0.92	1.2 (0.83 - 1.7)	0.33
BMI	0.6 (0.43 - 0.84)	0.0027	0.83 (0.6 - 1.1)	0.25
Differentiation	0.82 (0.57 - 1.2)	0.28	1.1 (0.73 - 1.5)	0.78
TNM stage	3.1 (2.1 - 4.6)	2.30E-08	4.4 (2.9 - 6.7)	1.30E-11
T_phase	3.9 (2.1 - 7.3)	1.60E-05	4 (2.2 - 7.5)	9.80E-06
N_phase	2 (1.7 - 2.5)	4.50E-11	2 (1.6 - 2.4)	4.50E-10
Tumor location	0.83 (0.68 - 1)	0.07	0.93 (0.76 - 1.1)	0.45
Tumor size	1.6 (1.1 - 2.2)	0.014	1.1 (0.77 - 1.6)	0.56
Chemotherapy	0.56 (0.39-0.81)	0.002	0.95 (0.67 - 1.4)	0.77
LDH_PA	3.7 (2.5 - 5.4)	5.70E-11	2.8 (1.9 - 4.1)	2.40E-07
LDH_ALB	4.5 (3 - 6.8)	6.80E-13	3.8 (2.5 - 5.7)	4.20E-10
GGT_PA	2.7 (1.8 - 3.9)	4.40E-07	2.2 (1.5 - 3.3)	5.50E-05
GGT_ALB	2.6 (1.7 - 3.9)	3.80E-06	2.6 (1.8 - 3.9)	2.30E-06
ALP_PA	3.2 (2.2 - 4.6)	1.60E-10	2.4 (1.7 - 3.5)	1.60E-06
ALP_ALB	2.3 (1.5 - 3.5)	0.00014	1.7 (1.1 - 2.5)	0.011
PLT_PA	2.5 (1.8 - 3.6)	2.70E-07	2 (1.4 - 2.9)	0.00011
PLT_ALB	2.1 (1.4 - 3)	0.00021	1.7 (1.1 - 2.5)	0.0093
LYM_PA	1.8 (1.3 - 2.6)	0.00095	1.3 (0.91 - 1.9)	0.15
LYM_ALB	0.64 (0.38 - 1.1)	0.085	0.59 (0.36 -0.96)	0.033
NEU_PA	2.5 (1.7 - 3.5)	6.10E-07	2.3 (1.6 - 3.3)	3.40E-06
NEU_ALB	2 (1.4 - 2.8)	0.00018	1.8 (1.3 - 2.6)	0.00091
ALB_CREA	0.5 (0.32 - 0.78)	0.0023	0.57 (0.36 - 0.9)	0.017
PA_CREA	0.32 (0.21- 0.49)	8.50E-08	0.46 (0.29-0.72)	0.00063
MON_PA	3 (2 - 4.6)	2.80E-07	2.7 (1.8 - 4.2)	3.90E-06
MON_ALB	3.4 (2.3 - 5.2)	4.60E-09	3.1 (2.1 - 4.7)	8.70E-08
GRIM_Score	3.1 (1.9 - 5.3)	1.80E-05	2.6 (1.5 - 4.5)	0.00043
PNI	0.31 (0.22- 0.45)	3.10E-10	0.44 (0.3 - 0.65)	2.50E-05
CONUT_score	1.7 (1.3 - 2.3)	5.60E-05	1.6 (1.2 - 2)	0.0012

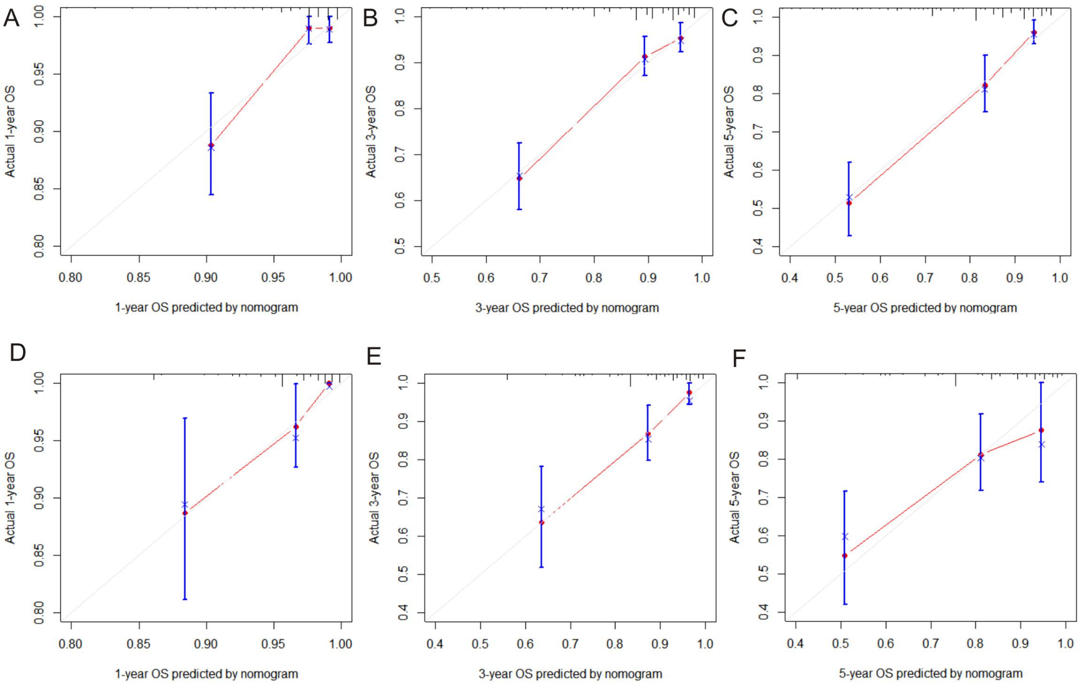

Figure S1. Calibration curves of OS-nomogram in the test (**A-C**) and validation cohorts (**D-F**).

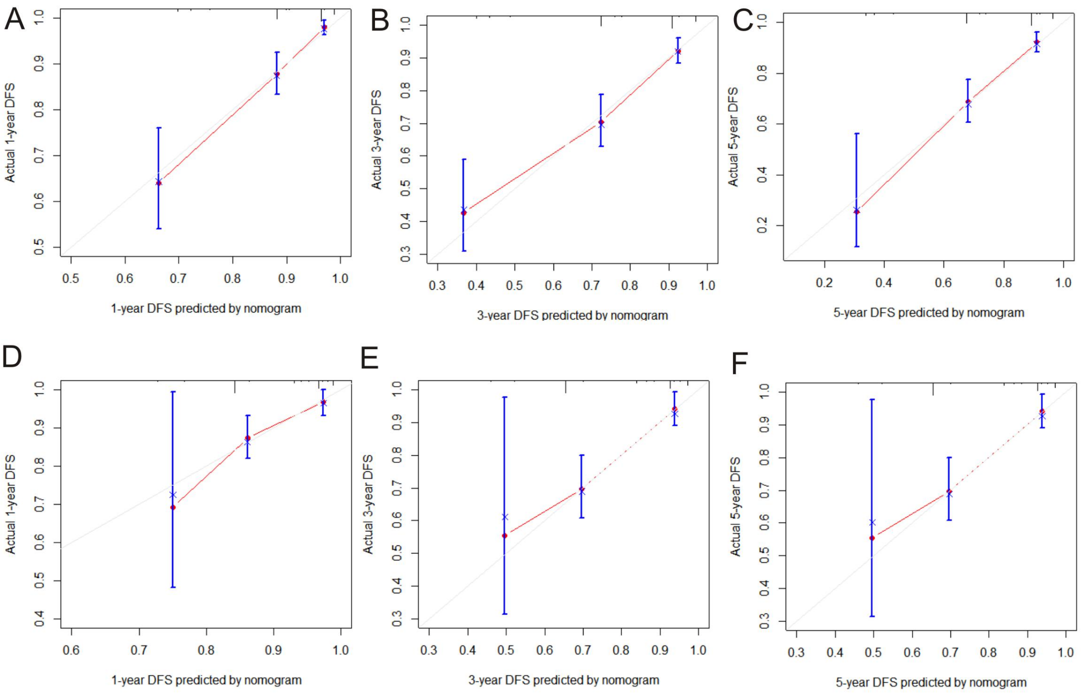

Figure S2. Calibration curves of DFS-nomogram in the test (**A-C**) and validation cohorts (**D-F**).
